# A Challenging View: Antibiotics Play a Role in the Regulation of the Energetic Metabolism of the Producing Bacteria

**DOI:** 10.3390/antibiotics9020083

**Published:** 2020-02-13

**Authors:** Marie-Joelle Virolle

**Affiliations:** Institute for Integrative Biology of the Cell (I2BC), Université Paris-Saclay, CEA, CNRS, 91198 Gif-sur-Yvette, France; marie-joelle.virolle@i2bc.paris-saclay.fr

**Keywords:** Streptomyces, antibiotics, energetic metabolism

## Abstract

Antibiotics are often considered as weapons conferring a competitive advantage to their producers in their ecological niche. However, since these molecules are produced in specific environmental conditions, notably phosphate limitation that triggers a specific metabolic state, they are likely to play important roles in the physiology of the producing bacteria that have been overlooked. Our recent experimental data as well as careful analysis of the scientific literature led us to propose that, in conditions of moderate to severe phosphate limitation—conditions known to generate energetic stress—antibiotics play crucial roles in the regulation of the energetic metabolism of the producing bacteria. A novel classification of antibiotics into types I, II, and III, based on the nature of the targets of these molecules and on their impact on the cellular physiology, is proposed. Type I antibiotics are known to target cellular membranes, inducing energy spilling and cell lysis of a fraction of the population to provide nutrients, and especially phosphate, to the surviving population. Type II antibiotics inhibit respiration through different strategies, to reduce ATP generation in conditions of low phosphate availability. Lastly, Type III antibiotics that are known to inhibit ATP consuming anabolic processes contribute to ATP saving in conditions of phosphate starvation.

## 1. Introduction

The Streptomyces genus is the most prolific producer of bioactive molecules that include numerous substances useful to human, animal, and plant health [1]. Some of these molecules have a signaling function and play a role in regulatory cascades leading to pathogenesis [2,3], but most of them have an antibiotic function [4]. It has long been known that the biosynthesis of most antibiotics takes place in the period of slow or no growth and that phosphate limitation is a major trigger of antibiotics biosynthesis [5]. Phosphate is not only a crucial element of cellular components such as membranous lipids and nucleic acids, it is also constitutive of the energetic molecules, ATP and polyphosphate (polyP). It is thus indispensable to most anabolic reactions and thus to life. In consequence, high and low extracellular concentration of Pi are correlated with high and low intracellular concentration of ATP and polyP [6]. Several reports indicate that when the concentration of Pi in the growth medium falls below a certain threshold (condition of Pi limitation), the bacteria triggers various adaptive responses to face this situation and restores its energetic balance [6,7]. One of the major responses to phosphate limitation is the induction of the expression of the two component system (TCS) PhoR/PhoP [7] that positively controls the expression of genes encoding proteins involved in the scavenging and uptake of phosphate (Pi) and negatively controls the expression of genes involved in the assimilation of nitrogen (N) [8,9]. Furthermore, some molecules signaling severe nutritional/energetic stress are also synthetized in these conditions. These include external signaling molecules such as those involved in quorum sensing (butyrolactone, homoserine lactone…) [10] and/or internal signaling molecules such as ppGpp, cyclic di GMP, etc. [11,12]. Numerous reports in the literature indicate that these molecules play a role in the triggering of antibiotic biosynthesis [13,14]. However, the links between a limitation in phosphate and the production of antibiotics remains poorly understood. In this Issue, a novel understanding of such links and novel functions of the antibiotics in the physiology of the producer are proposed. These proposals are based on recent experimental data from our group as well as on careful analysis of the abundant literature of the field.

## 2. Discussion

The model strain *S. coelicolor* is extensively used to study the regulation of antibiotic biosynthesis, since it produces abundantly three well characterized antibiotics: the colorless ionophore peptide Calcium Dependent Antibiotic CDA [15], the red pyrrole hybrid peptide/polyketide antibiotic undecylprodigiosin RED [16], and the blue pigmented polyketide antibiotic actinorhodin ACT [17]. The first antibiotic to be synthetized by *S. coelicolor*, in condition of phosphate limitation, is CDA [15]. The synthesis of CDA coincides with the transition phase, a phase of growth arrest characterized by extensive breakage of cellular macromolecules [18]. The production of RED [16,19] occurs a bit later, just before the entry into stationary phase, whereas that of ACT takes place in stationary phase [20].

CDA and RED are thought to create damage to the membrane leading to H+ gradient breakage and thus energy spilling, provoking cell death and lysis of a fraction of the population [21,22]. The cellular content of the lysed cells, released in the extracellular medium, is used to feed, and especially provide phosphate to, the surviving population. This phenomenon of programmed cell death (PCD) is well documented in *Streptomyces* [23,24], and the lysis of vegetative mycelium has long been considered as a prerequisite for the emergence of aerial mycelium [25]. Indeed, the possible role of CDA in PCD in SC has already been evoked [18], and that of RED was recently demonstrated [26]. Antibiotics fulfilling similar functions as CDA and RED are classified as Type I antibiotics. The supply of phosphate in conditions of low P availability is usually fulfilled by the induction of the expression of genes of the Pho regulon that are under the positive control of the two-component system PhoR/PhoP [8,9]. However, some of our recent work (Aaron-Millan Oropeza et al., submitted) demonstrated that the expression of PhoR/PhoP is much lower in *S. coelicolor* than in the phylogenetically closely related strain to *S. lividans* that does not produce antibiotics. This resulted in a high nitrogen but low phosphate availability in *S. coelicolor* compared to *S. lividans*. Consequently, in order to fulfill its phosphate needs, *S. coelicolor* adopts an alternative strategy that is the triggering of the biosynthesis of type I antibiotics. The phosphate resulting from cell lysis allows the restoration of the energy balance of the cell *via* the activation of its oxidative metabolism [6]. The activation of the oxidative metabolism requires the enhanced expression and stimulation of the activity of the enzymes of the Krebs cycle generating energy (GTP) or reduced cofactors (NADH, FADH2) whose re-oxidation by the respiratory chain generates ATP (Aaron-Millan Oropeza et al., submitted). The necessary fuelling of the Krebs cycle by acetylCoA, in *S. coelicolor*, is correlated with a reduced synthesis of storage lipids of the TriAcylGlycerol (TAG) family that also requires acetylCoA for its synthesis [6].

However, when Pi becomes too scarce to support the ATP generating function of the respiratory chain, a blockage of the latter occurs, resulting in electron leakage toward secondary acceptors and thus generation of oxidative stress (ROS/RNS). Many reports in the literature suggest that oxidative stress might be an important trigger of the biosynthesis of some antibiotics in *Streptomyces* as in fungi [27,28,29,30], called “Type II” antibiotics. Some of these antibiotics, including ACT, contain quinone groups that are able to capture electrons [31]. They have thus antioxidant properties, and their biosynthesis could be considered as an adaptive response to oxidative stress (for example, chromomycin [32]). Furthermore, and most importantly, these molecules also have the ability to reduce the electron flow in the respiratory chain, leading to lower efficiency of the latter and thus to a reduction of ATP generation. Indeed, we previously demonstrated that the onset of ACT biosynthesis coincides with an abrupt drop in the intracellular ATP concentration in *S. coelicolor* [6] and that the deletion of the ACT cluster is correlated with an enhanced sensitivity to oxidative stress (Aaron-Millan Oropeza et al., submitted). Other type II antibiotics achieve a similar reduction of ATP generation by targeting different components of the respiratory chain [33,34,35]. For instance, oligomycin inhibits ATP synthase activity *via* the blockage of H+ entry [36,37] whereas antimycin inhibits the cytochrome c reductase of the electron transport chain [38]. The function of “type II” antibiotics is to reduce the efficiency of respiration in order to adjust ATP synthesis to low Pi availability.

At last, if ATP cannot be generated because of extreme Pi scarcity, it has to be saved to maintain an energy balance compatible with the survival of bacteria. In that case of severe energy stress, the biosynthesis of another type of antibiotics called “Type III” is triggered. Type III antibiotics are known to specifically inhibit ATP consuming anabolic processes, such as the biosynthesis of DNA, RNA, proteins, membrane lipids, and cell walls. They can thus contribute to energy saving. The signal triggering the biosynthesis of type III antibiotics might be a high concentration of NMP, which signals a severe energy deficit, as in eukaryotic microorganisms [39]. “Type III” antibiotics are the most well known antibiotics that are widely used in medicine. They are less toxic than type I or II antibiotics, which target membranes or respiration, since they target specific bacterial/fungal machineries (https://www.orthobullets.com/basic-science/9059/antibiotic-classification-and-mechanism).

## 3. Conclusions

**Figure 1:**
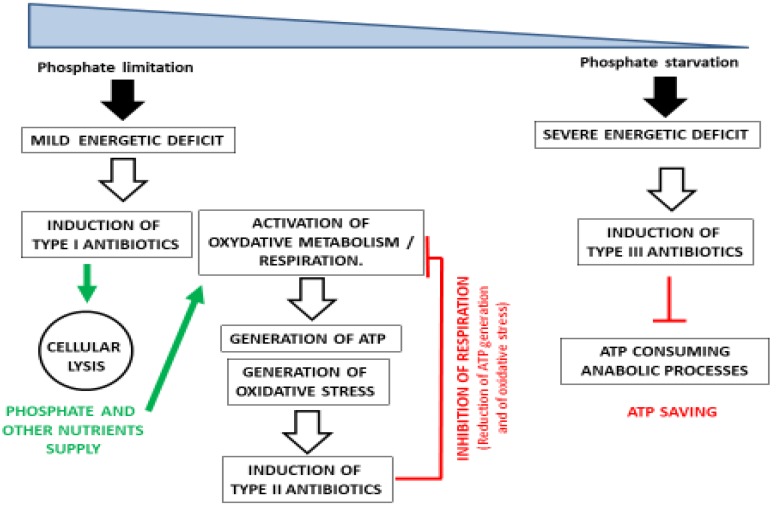
Schematic representation of the role played by each type of antibiotic in the regulation of the energy metabolism of Streptomyces in condition of moderate to severe phosphate limitation.

In summary, the three types of antibiotics mentioned above contribute, through different strategies, to regulate cellular energy either *via* the supply of phosphate and other nutrients due to cell lysis and autophagy (type I), to the inhibition of ATP generation (Type II) and to ATP saving (Type III) (Figure 1). However, we cannot totally exclude that the synthesis of these molecules in itself also contributes to the metabolic adjustment of the bacteria facing growth reduction for whatever reasons (and they can be multiple). Indeed, in the condition of growth reduction, the amount of metabolic intermediates, ATP, and reducing power generated by catabolism might exceed the needs of anabolism. Consequently, high ATP content might paradoxically be a consequence and signals growth slow down. The accumulation of these molecules could have detrimental osmotic or regulatory impacts on the strain, and their consumption for the biosynthesis of secondary metabolites could attenuate these negative effects [40]. This constitutes the well-known hypothesis of secondary metabolites such as metabolic sink.

These bioactive molecules are toxic for their producer as well as for other micro-organisms, but the very strict temporal control of their biosynthesis and the induction of resistance determinants (excretion of the antibiotic, modification of the antibiotic or of its target, etc…) could limit their toxicity to a very short period in the producer [41,42]. These toxic molecules are often excreted into the environment, where they are assumed to limit growth or kill microorganisms present in the same ecological niche as *Streptomyces* [43]. These environmental microorganisms are likely to be more sensitive than the *Streptomyces* to these harmful molecules, since they do not possess similar resistance mechanisms. These molecules thus confer a competitive advantage to their producer that can then monopolize the limited nutritious resources present in their ecological niche or even feed on the cellular constituents of their lysed competitors [44]. The proposed endogenous role played by the antibiotics in the regulation of the energetic metabolism of the producing bacteria, and their exogenous role of warfare traditionally mentioned [45], are not mutually exclusive, nor contradictory; they simply constitute the two sides of the same coin.
